# α-Synuclein Up-regulates Monoamine Oxidase A Expression and Activity *via* Trans-Acting Transcription Factor 1

**DOI:** 10.3389/fnagi.2021.653379

**Published:** 2021-03-17

**Authors:** Congcong Jia, Cheng Cheng, Tianbai Li, Xi Chen, Yuting Yang, Xinyao Liu, Song Li, Weidong Le

**Affiliations:** ^1^Center for Clinical Research on Neurological Diseases, The First Affiliated Hospital, Dalian Medical University, Dalian, China; ^2^Liaoning Provincial Key Laboratory for Research on the Pathogenic Mechanisms of Neurological Diseases, The First Affiliated Hospital, Dalian Medical University, Dalian, China; ^3^Department and Institute of Neurology, Sichuan Academy of Medical Sciences, Sichuan Provincial Hospital, Chengdu, China

**Keywords:** α-Synuclein, MAOA, Parkinson’s disease, Sp1, dopamine

## Abstract

Abnormal α-Synuclein (α-SYN) aggregates are the pathological hallmarks of Parkinson’s disease (PD), which may affect dopamine (DA) neuron function and DA metabolism. Monoamine oxidase A (MAOA) is an enzyme located on the outer mitochondrial membrane that catalyzes the oxidative deamination of DA. Both α-SYN and MAOA are associated with PD pathogenesis, suggesting possible crosstalk between these two molecules. In the present study, we aimed to investigate the potential impacts of α-SYN on MAOA function and further explore the underlying mechanisms. Our study showed that overexpression of α-SYN [both wild-type (WT) and A53T] increased MAOA function *via* upregulating its expression without impacting MAOA stability. Overexpression of α-SYN^WT^ or α-SYN^A53T^ enhanced the transcription activity of the MAOA promoter region containing the binding sites of cell division cycle associated 7 like (R1, a transcriptional repressor of MAOA) and trans-acting transcription factor 1 (Sp1, a transcription factor of MAOA). Interestingly, α-SYN selectively increased Sp1 expression, thereby enhancing the binding capacity of Sp1 with MAOA promoter to increase MAOA expression. Taken together, our findings demonstrate that α-SYN can upregulate MAOA expression *via* modulation of Sp1 and may shed light on future studies of α-SYN associated PD pathogenesis.

## Introduction

Parkinson’s disease (PD) is a neurodegenerative disease pathologically characterized by progressive loss of dopamine (DA) neurons in the striatum and substantia nigra (Lees et al., 2009). Genetic alterations, such as the reduction or mutation of genes, can cause familial forms of PD (Kalia and Lang, 2015). Among them, α-Synuclein (α-SYN) gene (SNCA) was the first gene linked to PD. α-SYN is the principal constituent of Lewy bodies, and variation at its locus is the major genetic risk factor for sporadic disease. Polymeropoulos et al. (1997) identified the G209A mutation of SNCA, resulting in a substitution of Ala at position 53 to Thr (A53T), in familial forms of PD. Until now, there are six missense mutations of α-SYN (A53T, A30P, A53E, E46K, G51D, and H50Q) associated with PD (Polymeropoulos et al., 1997; Kruger et al., 1998; Zarranz et al., 2004; Appel-Cresswell et al., 2013; Lesage et al., 2013; Proukakis et al., 2013). Mutations of α-SYN can form toxic protofibril or fibril (Giasson et al., 2002), affecting the functions of DA neurons and inducing neurodegeneration (Dev et al., 2003). Previous studies have shown that α-SYN affects the expressions of DA-related genes *via* Nurr1, a key factor involved in the development and functional maintenance of DA neurons (Saucedo-Cardenas et al., 1998; Jankovic et al., 2005; Jia et al., 2020a). Besides, α-SYN accumulation is also associated with tau spreading synaptic vesicles, autophagy, mitochondria, endoplasmic reticulum and Golgi complex, nucleus, cell-to-cell propagation, and neuroinflammation related functions (Wong and Krainc, 2017; Bassil et al., 2021). All these findings indicate the important functions of α-SYN in PD pathogenesis.

Monoamine oxidase (MAO), an enzyme for biogenic amines degradation, is located on the outer membrane of mitochondria (Green and Youdim, 1975). MAO has two isoforms (MAOA and MAOB), which locate on adjacent homologous genes of the X chromosome (Shih, 1991). While MAOA is a critical regulator of the metabolisms of serotonin (5-HT) and norepinephrine (NE), and MAOB prefers benzylamine and phenethylamine (Shih et al., 1999), both of them are involved in DA metabolism in most species (Westlund et al., 1988; Ugun-Klusek et al., 2019). Moreover, both MAOA and MAOB transcription levels and enzymatic activities are significantly increased in the induced pluripotent stem cells of dermal fibroblasts from PD patients with parkin mutations (Jiang et al., 2012). Besides, MAOA is also involved in apoptotic signaling and Shh-IL6-Rankl signaling pathways (Ou et al., 2006; Wu et al., 2017), indicating important roles of MAOA in DA neuron loss of PD. All these findings suggest the close association of MAO with PD pathogenesis.

Interestingly, the DA level was significantly reduced in the striatum tissue of mice with DA neurons specifically overexpressed human α-SYN-A53T (Chen et al., 2015), and the release of DA in the dorsal striatum of 1-month-old A53T mice is also reduced (Lin et al., 2012). Although α-SYN plays an important role in the DA-associated pathogenesis of PD, the molecular mechanism of α-SYN regulation on DA metabolism is still far from being clearly understood. One previous study reported that α-SYN can directly bind with MAOB and enhance MAOB enzyme activity (Kang et al., 2018). However, whether α-SYN modulates the expression and function of MAOA is still rarely investigated. In this study, we demonstrated that MAOA was up-regulated and activated in α-SYN overexpressed cells through increasing its transcription factor (Sp1) expression and the binding capacity of Sp1 with the MAOA promoter region. Our findings may shed light on future studies of α-SYN associated PD pathogenesis.

## Materials and Methods

### Cell Lines and Culture

Mouse neuroblastoma N2a cells were maintained in Dulbecco’s modified Eagle’s medium (Gibco, Gaithersburg, MD, USA) containing 1% penicillin/streptomycin solution (Sigma–Aldrich, St. Louis, MO, USA) and 10% fetal bovine serum (Gibco). For the establishment of N2a cell lines stably expressing either wild-type (WT) α-SYN (N2a/α-SYN^WT^) or A53T mutant α-SYN (N2a/α-SYN^A53T^), N2a cells were transfected with p3XFLAG-CMV10, p3XFLAG-CMV10-human-SYNC, or p3XFLAG-CMV10-human-SYNC-A53T plasmids (kindly gift by Huaibin Cai professor) by lipofectamine 6000 (Beyotime, Shanghai, China) according to the manufacturer’s instructions. Forty-eight hours after transfection, the cells were screened by cell culture medium containing 600 μg/ml of G418 (Sigma–Aldrich) until no more cell death. Real-time PCR and Western blot were used to verify the α-SYN level in cells.

### Reagents

The antibodies used in the present study were shown as follows: anti-α-SYN antibody (Santa Cruz Biotechnology, Dallas, TX, USA), anti-MAOA antibody (Abcam, Cambridge, MA, USA), anti-Sp1 (Abcam, Cambridge, MA, USA), anti-R1 (ABclonal Technology, Wuhan, China), anti-Histone antibody (Cell Signaling Technology), anti-GAPDH antibody (Cell Signaling Technology, Danvers, MA, USA), anti-MAOB, anti-COMT, anti-ADH5, anti-FLAG, Goat anti-mouse IgG H&L and Goat anti-rabbit IgG H&L (Proteintech Group Inc., Rosemont, IL, USA), Goat anti-mouse Alexa 594 (Abcam) and Goat anti-rabbit Alexa 488 (Cell Signaling Technology).

### Western Blot

N2a/α-SYN and N2a control cells were resuspended in RIPA lysis buffer (Beyotime) containing a protease inhibitor (cocktail, Sigma–Aldrich), lysed on ice for 30 min, and centrifuged at 12,000× *g* for 15 min. The supernatant protein concentration was detected by a BCA Protein Assay kit (Beyotime). Samples with equal amounts of total proteins (30–50 μg) were subjected to 10 or 12.5% SDS-polyacrylamide gel electrophoresis and then transferred to 0.45 μm polyvinylidene difluoride membranes (Millipore, Burlington, MA, USA). After blocking with 5% skimmed milk for 1 h at room temperature, the membrane was incubated with the primary antibody at 4°C overnight. Then, the membrane was incubated with a secondary antibody, and the protein bands were detected with the enhanced chemiluminescence detection kit (Wanlei Biotechnology, Shenyang, China) and quantified using the FluorChem Q system (Protein Simple, San Jose, CA, USA) and normalized against reference protein values.

### Real-Time PCR

Total RNA was extracted and used to synthesize cDNA according to the manufacturer’s instructions (TransGen Biotechnology, Beijing, China). The primers sequences and analysis methods used in the present study were described previously (Jia et al., 2020a, b).

### Immunofluorescence

N2a/α-SYN and N2a control cells were fixed with 4% paraformaldehyde, punched by 0.2% Triton X-100 and blocked with 2% bovine serum albumin for 30 min, followed by incubation with the primary antibodies overnight at 4°C. After washing with PBS, the cells were incubated with fluorescent secondary antibodies at room temperature for 1 h. Antifade Mounting Medium with DAPI (Beyotime) was used for nuclei staining. The cells were then washed with PBS three times. Fluorescence images were visualized using an A1R MP multiphoton confocal microscope (Nikon, Tokyo, Japan).

### Immunoenzymatic Measurement

The samples were rinsed with ice-cold PBS and sonicated in PBS containing a protease inhibitor cocktail (Sigma–Aldrich). The homogenates were centrifugated for 10 min at 5,000× *g* to get the supernatant. Equal amounts of total supernatant protein were used to detect MAOA enzyme activity and DA content, using a mouse MAOA ELISA kit and a DA ELISA kit respectively, according to the manufacturer’s instructions (Dalian Aimoke Biotechnology Company Limited, Dalian, China).

### Luciferase Reporter Assay

N2a/α-SYN and N2a control cells were co-transfected with MAOA promoter fragment luciferase reporter plasmid (0.5 μg) and phRL-SV40 vector (0.05 μg) using lipofectamine 6000 (Beyotime) for 48 h according to our precious protocol (Jia et al., 2020b). Cells were sonicated in lysis buffer and then centrifuged for 10 min at 12,000× *g* to get the supernatant. The luciferase activities of the supernatant were detected with a dual-luciferase assay kit (Beyotime) in a microplate reader (BioTek Instruments, Inc., VT, USA) according to the manufacturer’s instructions. The value of firefly luciferase was normalized to Renilla luciferase.

### Chromatin Immunoprecipitation

A ChIP assay kit (Beyotime) was used to detect the binding amount of Sp1 or R1 with the MAOA promoter. Briefly, cells were crosslinked with 1% formaldehyde, sonicated with PBS containing 1 mM phenylmethylsulfonyl fluoride, and centrifuged at 12,000× *g* for 5 min. Equal amounts of total proteins were added anti-IgG, anti-R1, and anti-Sp1 antibodies, respectively. On the second day, protein A/G beads were added to bind the antibody-target protein-DNA complex. The precipitated complexes were washed and eluted to obtain the enriched target protein-DNA complex. The obtained complexes were purified with a DNA purification kit (Beyotime) and then detected with Real-time PCR. The methods of analyzed values were described previously (Jia et al., 2020a, b).

### Immunoprecipitation

We seeded α-SYN overexpressed and control cells in 10 cm plates. The next day, the samples were washed with PBS, lysed in IP lysis buffer (Beyotime) containing cocktail, and centrifuged at 12,000× *g* for 15 min. Anti-FLAG magnetic beads (MedChemExpress LLC, Monmouth Junction, NJ, USA) were added into equal amounts of total proteins to make antibody-FLAG-protein complex. The complex was washed with PBS and then eluted in 1× SDS loading buffer for Western blot.

### Statistical Analysis

All experiments were performed in triplicate and repeated a minimum of three times. Data were expressed as mean ± SEM. Statistical significance for multiple comparisons was performed by one-way analysis of variance (ANOVA) using GraphPad Prism (version 8.0.2, GraphPad Inc., San Diego, CA, USA). The significance was considered at *p* < 0.05.

## Results

### Overexpression of α-SYN Decreases DA Level and Increases MAOA Activity

**Figure 1:**
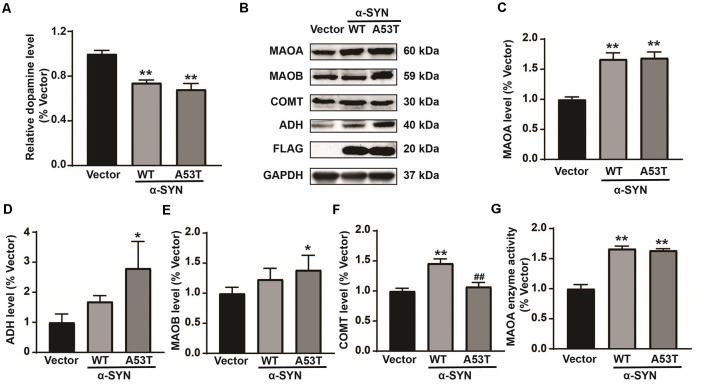
Impacts of α-SYN^WT^ and α-SYN^A53T^ on dopamine (DA) content and DA metabolism-associated enzymes. The DA content was detected in α-Synuclein (α-SYN) [wild-type (WT), A53T] stably overexpressed and control (Vector) cells with a mouse DA Elisa kit **(A)**. Western blot detected the protein level of DA metabolism-associated enzymes [Monoamine oxidase A (MAOA), Monoamine oxidase B (MAOB), catechol O-methyltransferase (COMT), and alcohol dehydrogenase (ADH)] **(B–F)**. MAOA enzyme activity was detected in α-SYN overexpressed cells with a mouse MAOA Elisa kit **(G)**. Data were expressed as mean ± SEM. **p < 0.01 vs. Vector transfected cells, *p < 0.05 vs. Vector transfected cells, ^##^*p* < 0.01 vs. α-SYN-WT transfected cells, *n* = 3.

To explore the mechanism underlying the impacts of α-SYN on DA metabolism, we first constructed α-SYN^WT^ and α-SYN^A53T^ stably overexpressed N2a cell lines and detected the level of DA in these cells with a DA ELISA kit. The results showed that overexpression of α-SYN (both WT and A53T) induced 26% and 32% DA reduction for WT and A53T respectively, compared with control groups (*p* < 0.01, Figure 1A). We then detected the expression levels of several key proteins associated with DA metabolism, including MAOA, MAOB, alcohol dehydrogenase (ADH), and catechol O-methyltransferase (COMT). Western blot analysis showed that, among these proteins, MAOA was most sensitive to overexpression of α-SYN^WT^ and α-SYN^A53T^, as evidenced by 1.67 and 1.69 folds upregulation, respectively (*p* < 0.01, Figures 1B,C). Other proteins (MAOB, ADH, and COMT) were not significantly changed by both α-SYN^WT^ and α-SYN^A53T^ overexpression (Figures 1B,D–F). Therefore, MAOA was selected for further analysis. We determined the total MAOA activity of cell lysates from α-SYN^WT^ and α-SYN^A53T^ overexpressed cells using a mouse MAOA ELISA kit. Consistent with protein expressions, the results showed that overexpression of α-SYN^WT^ and α-SYN^A53T^ markedly increased MAOA enzyme activity to 1.66 and 1.64 folds of controls (*p* < 0.01, Figure 1G). All these data indicated that overexpression of α-SYN^WT^ and α-SYN^A53T^ increased MAOA expression and activity, further confirming the association of α-SYN and PD pathology.

### α-SYN Neither Binds With MAOA nor Alters the Degradation of MAOA

**Figure 2:**
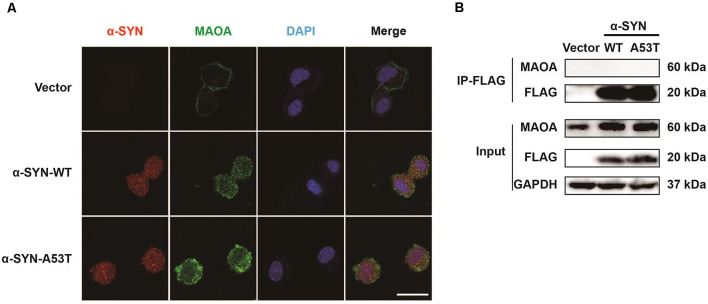
Colocalization and interaction of α-SYN and MAOA. The intracellular protein level of MAOA was detected using immunofluorescent staining, α-SYN (red), MAOA (green), and nucleus (blue). Scale bare: 20 μm **(A)**. The transfected α-SYN was pulled down with an anti-FLAG antibody, and the direct binding between α-SYN and MAOA was detected in the control cells (Vector), α-SYN-WT and α-SYN-A53T stable expression cells **(B)**.

We previously found that α-SYN is located in both the nucleus and cytoplasm (Jia et al., 2020a). MAOA is mainly expressed on the outer membrane of mitochondria (Green and Youdim, 1975). To explore the mechanism of α-SYN on MAOA expression, we used the immunofluorescence staining to detect the colocalization of α-SYN and MAOA. As expected, our results showed that MAOA expression was increased in α-SYN^WT^ and α-SYN^A53T^ overexpressed cells (Figure 2A). Moreover, α-SYN showed clear colocalizations with MAOA in the cytoplasm. Due to the α-SYN^WT^ and α-SYN^A53T^ fragments of plasmids fused with a 3XFLAG label protein, we used an anti-FLAG antibody to pull down the transfected α-SYN to detect the direct binding between α-SYN and MAOA in the above cells. Interestingly, while α-SYN and MAOA colocalized in the cytoplasm (Figure 2A), no direct binding was observed between α-SYN and MAOA (Figure 2B).

**Figure 3:**
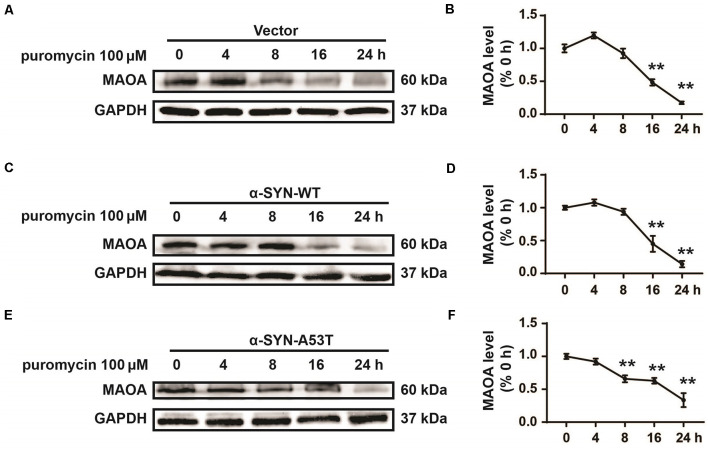
Effects of overexpression of α-SYN^WT^ and α-SYN^A53T^ on MAOA degradation. The protein level of MAOA was detected using Western blot in α-SYN (WT and A53T) stably overexpressed and control (Vector) cells cultured with puromycin (100 μM) for 0, 4, 8, 16, and 24 h **(A–F)**. Data were expressed as mean ± SEM. **p < 0.01 vs. the same cells at 0 h point, *n* = 3.

One possibility for the increased level of MAOA in N2a/α-SYN cells might be due to the inhibited MAOA degradation. To further confirm this possibility, we cultured α-SYN^WT^ and α-SYN^A53T^ overexpressed cells with a protein synthesis inhibitor puromycin (100 μM) for 0, 4, 8, 16, and 24 h. MAOA expression was determined by Western blot. The results showed that the half-life degradation of MAOA in N2a/α-SYN (WT and A53T) cells were not significantly different from that in the control cells (Figures 3A–F). These results indicated that α-SYN up-regulated MAOA expression not *via* inhibiting MAOA degradation.

### α-SYN Affects the Protein Synthesis of MAOA *via* Sp1

**Figure 4:**
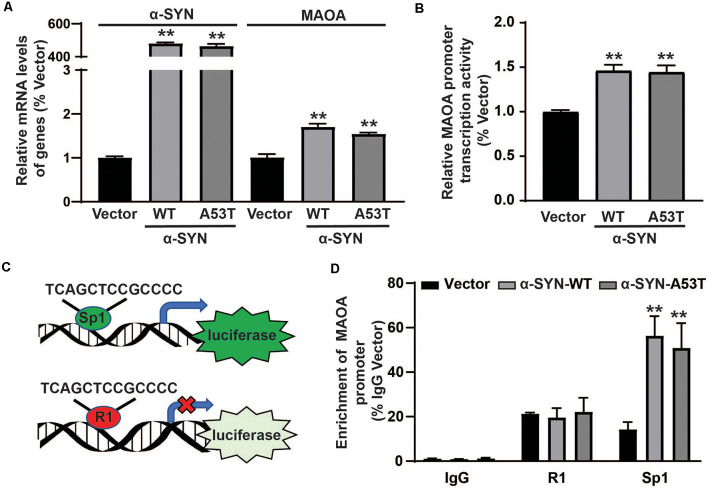
α-SYN affected the protein synthesis of MAOA. The mRNA level of MAOA **(A)** and the activity of MAOA promoter luciferase **(B)** were analyzed in control cells (Vector), α-SYN^WT^ and α-SYN^A53T^ stably overexpressed cells. The transcription factor diagram of bioinformatics analysis MAOA promoter luciferase plasmid **(C)**. ChIP-Real-time PCR analyzed the binding quantity of R1 and Sp1 with the MAOA promoter region **(D)**. Data were expressed as mean ± SEM. **p < 0.01 vs. Vector transfected cells, *n* = 3.

After ruling out the possibility of direct effects of α-SYN on MAOA degradation, we detected the mRNA level of MAOA in α-SYN^WT^ and α-SYN^A53T^ overexpressed cells. Real-time PCR results showed that, together with significantly increased α-SYN mRNA level, MAOA level was up-regulated to 1.69 and 1.54-folds by α-SYN overexpression (*p* < 0.01, Figure 4A), indicating α-SYN altered the synthesis of MAOA from mRNA level. To further explore the mechanism, we subcloned the core fragment of the MAOA promoter into the pGL3-basic vector to obtain the MAOApromoter-luciferase plasmid, co-transfected with phRL-SV40 plasmids into α-SYN^WT^ and α-SYN^A53T^ overexpressed cells. The results of dual-luciferase reporter gene assay showed that both α-SYN^WT^ and α-SYN^A53T^ overexpression up-regulated the activity of MAOA-luciferase plasmid by 1.46 and 1.44 folds, respectively (*p* < 0.01, Figure 4B). We then used the JASPAR database to analyze the MAOA core promoter region of luciferase plasmid (Fornes et al., 2020). Bioinformatics analysis showed that the region contained the same binding sites of cell division cycle associated 7 like (R1, a transcriptional repressor of MAOA) and Sp1 (a transcription factor of MAOA; Figure 4C). α-SYN may affect the balance of R1 and Sp1 binding with MAOA promoter to impact MAOA expression. ChIP-Real-time PCR results showed that α-SYN did not significantly change the binding quantity of R1 with the MAOA promoter but increased that of Sp1 to 3.95 folds and 3.57 folds compared with controls, respectively (*p* < 0.01, Figure 4D).

**Figure 5:**
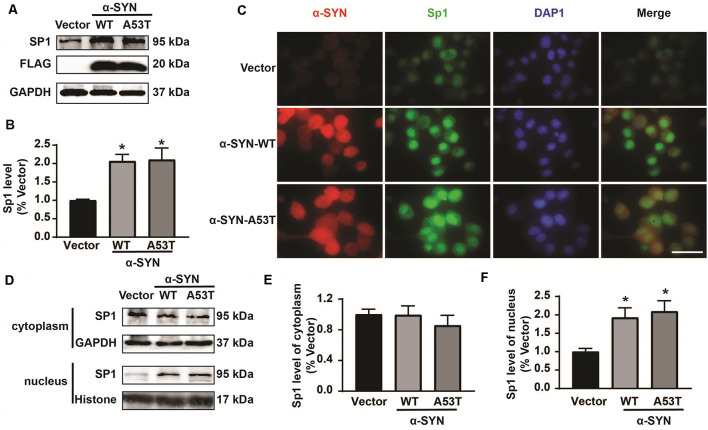
α-SYN regulated the expression of Sp1. Western blot was used to detect the protein level of Sp1 in control cells (Vector), α-SYN^WT^ and α-SYN^A53T^ stably overexpressed cells **(A,B)**. The intracellular protein level of Sp1 was confirmed using immunofluorescent staining, α-SYN (red), Sp1 (green), and nucleus (blue). Scale bare: 50 μm **(C)**. Western blot detected the cytoplasm and nucleus protein level of Sp1 in control cells (Vector), α-SYN^WT^ and α-SYN^A53T^ stably overexpressed cells **(D–F)**. Data were expressed as mean ± SEM. *p < 0.05 vs. Vector transfected cells, *n* = 3.

**Figure 6:**
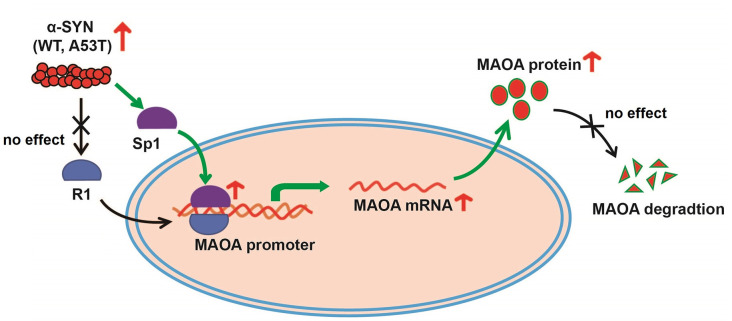
The schematic illustration of mechanisms underlying α-SYN-induced MAOA increase. Overexpression of α-SYN^WT^ and α-SYN^A53T^ selectively up-regulated Sp1 expression and the binding quantity of Sp1 with MAOA promoter, thereby increasing MAOA expression and activity.

Then, we determined the total protein levels of Sp1 in α-SYN^WT^ and α-SYN^A53T^ overexpressed cells, the results showed that α-SYN increased Sp1 expression to 2.06 folds and 2.10 folds compared with controls (*p* < 0.05, Figures 5A,B). Consistently, immunofluorescence staining obtained the same results as Western blot, confirming the significant increase in Sp1 expression in the nucleus of α-SYN overexpressed cells (Figure 5C). We separated cytoplasm-nucleus proteins and detected the expression levels of Sp1. Western blot results indicated that α-SYN did not significantly change the cytoplasm protein level of Sp1 (Figures 5D,E), it selectively increased the nucleus protein level of Sp1 to 1.92 folds and 2.10 folds compared with controls (*p* < 0.05, Figures 5D,F). Altogether, our results demonstrate that α-SYN can increase MAOA protein synthesis *via* up-regulating Sp1 expression (Figure 6).

## Discussion

α-SYN, as the important components of Lewy bodies, is closely associated with DA neuron loss and DA level reduction in the PD brain (Spillantini et al., 1997). One previous study showed that substantia nigra injection of AAV1/2-α-SYN^A53T^ reduced striatal DA and DOPAC levels and increased HVA levels (Ip et al., 2017). The dorsolateral striatum of α-SYN^A53T^ mice demonstrates a lower DA level than those of WT controls (Lin et al., 2012). In this study, we also found that overexpression of α-SYN (both WT and A53T) reduced DA contents *in vitro* (Figure 1A). These findings suggested a close correlation between α-SYN and DA metabolism. However, the exact mechanisms for this pathological impact of α-SYN on DA biosynthesis or degradation are still far from being fully investigated.

As for the biosynthesis of DA, L-tyrosine is hydroxylated by tyrosine hydroxylase (TH) to generate L-DOPA and then decarboxylated by aromatic acid decarboxylase (AADC) to form DA (Daubner et al., 2011; Monzani et al., 2019). Our previous study showed that α-SYN down-regulated the expression levels of TH and AADC *via* suppressing Nurr1 gene expression (Jia et al., 2020a), indicating an impact of α-SYN on DA biosynthesis through Nurr1-related mechanisms. In this study, we further explored the potential impacts and molecular mechanism of α-SYN on DA degradation. A previous study found that α-SYN can directly bind to MAOB and increase its activity and expression (Kang et al., 2018). Consistently, our present data also revealed that overexpression of α-SYN^A53T^ significantly up-regulated MAOB expression (Figures 1B,E). Except for MAOB, our results further suggested that α-SYN changed the expression levels of MAOA, COMT, and ADH (Figures 1B–D,F). Much more interestingly, among these proteins, MAOA was the only one that was significantly up-regulated by both α-SYN^WT^ and α-SYN^A53T^ overexpression, indicating an important role of MAOA in the α-SYN-regulated DA degradation.

To explore the mechanism of α-SYN on MAOA expression, we detected the co-localization of α-SYN and MAOA and the data revealed that there was no direct interaction between α-SYN and MAOA, which is consistent with previous findings (Kang et al., 2018). Our previous study and others showed that MAOA was degraded *in vitro* with the presence of a protein synthesis inhibitor (Jiang et al., 2006; Jia et al., 2020b). Kabayama et al. found that RING finger-type E3 ubiquitin ligase Rines/RNF180 increased the ubiquitination and degradation of MAOA (Kabayama et al., 2013). Moreover, the A30P mutation of α-SYN induced the activity of proteasome (Tanaka et al., 2001). α-SYN promotes the proteasome-dependent degradation of Nurr1 expression (Lin et al., 2012). These results raised the possibility that α-SYN up-regulated MAOA expression partially *via* changing the proteasome-dependent posttranscriptional levels. However, in our present study, both α-SYN^WT^ and α-SYN^A53T^ failed to induce a significant change in the half-life of MAOA protein (Figure 3). Our findings suggest possible transcription mechanisms underlying the impacts of α-SYN-increased MAOA level. In agreement with this hypothesis, our results showed that α-SYN increased MAOA mRNA level and the promoter activity (Figures 4A,B).

Previous studies reported that the human MAOA core promoter region has clusters of Sp1 binding sites, which can be competitively bound by R1 to repress MAOA expression (Zhu et al., 1994; Chen et al., 2005). The balance between Sp1 transcriptional activity and R1 repressor activity on the promoter regulates MAOA expression (Huang et al., 2012). Our bioinformatics analysis showed that the MAOA promoter-luciferase plasmid contained the binding site of Sp1 and R1. ChIP-Real-time PCR results indicated that α-SYN selectively increased the binding quality of Sp1 with MAOA promoter, without change that of R1. Rather intriguingly, the protein level of Sp1, especially the nuclear Sp1, was increased by α-SYN overexpression. Our study showed that both α-SYN^WT^ and α-SYN^A53T^ can increase the activity and expression of MAOA *via* Sp1, suggesting that the increase of MAOA by α-SYN is one of the important molecular determinants for abnormal metabolism of DA in PD.

MAOA, as a flavoenzyme, can catabolize other neurotransmitters, including NE and 5-HT (Shih et al., 1999). The dysregulated functions of MAOA in neural signal transmission may lead to depression, autism, or aggressive behavior phenotypes (Gu et al., 2017; Kolla and Bortolato, 2020), which may be involved as non-motor psychiatric manifestations of PD. Moreover, bypass of melatonin synthesis, MAOA converts 5-HT into 5-hydroxyindoleacetic acid (5-HIAA; Reiter, 1991), which may lead to a sleep disorder, another type of non-motor symptom of PD. These findings further predict the possible involvement of MAOA in non-motor symptoms of PD. Our results may provide a basis for studying the mechanism of α-SYN on the non-motor symptom of PD.

In summary, our present data reveal that α-SYN induces DA content *via* the effect on Sp1-mediated MAOA synthesis and activity, the finding of which is unknown for the α-SYN association with MAOA and its possible link to PD. Our study may shed new light on future studies to uncover the molecular mechanism of α-SYN on DA metabolism in PD pathogenesis and to facilitate the exploration of molecular targets for PD therapy.

## Data Availability Statement

The raw data supporting the conclusions of this article will be made available by the authors, without undue reservation.

## Author Contributions

CJ, CC, and TL conducted the experiments. CJ, XC, YY, and XL collected and analyzed the data. CJ and WL drafted the manuscript. WL and SL revised the manuscript. WL conceived and designed the experiments. The final manuscript was read and approved by all authors. All authors contributed to the article and approved the submitted version.

## Conflict of Interest

The authors declare that the research was conducted in the absence of any commercial or financial relationships that could be construed as a potential conflict of interest.

## References

[B1] Appel-Cresswell S. Vilarino-Guell C. Encarnacion M. Sherman H. Yu I. Shah B.. (2013). α-synuclein p.H50Q, a novel pathogenic mutation for Parkinson’s disease. Mov. Disord. 28, 811–813. 10.1002/mds.25421 23457019

[B2] Bassil F. Meymand E. S. Brown H. J. Xu H. Cox T. O. Pattabhiraman S.. (2021). α-Synuclein modulates tau spreading in mouse brains. J. Exp. Med. 218:e20192193. 10.1084/jem.20192193 33091110 PMC7588140

[B3] Chen K. Ou X. M. Chen G. Choi S. H. Shih J. C. (2005). R1, a novel repressor of the human monoamine oxidase A. J. Biol. Chem. 280, 11552–11559. 10.1074/jbc.M410033200 15654081 PMC2861901

[B4] Chen L. Xie Z. Turkson S. Zhuang X. (2015). A53T human α-synuclein overexpression in transgenic mice induces pervasive mitochondria macroautophagy defects preceding dopamine neuron degeneration. J. Neurosci. 35, 890–905. 10.1523/JNEUROSCI.0089-14.2015 25609609 PMC4300331

[B5] Daubner S. C. Le T. Wang S. (2011). Tyrosine hydroxylase and regulation of dopamine synthesis. Arch. Biochem. Biophys. 508, 1–12. 10.1016/j.abb.2010.12.017 21176768 PMC3065393

[B6] Dev K. K. Hofele K. Barbieri S. Buchman V. L. van der Putten H. (2003). Part II: α-synuclein and its molecular pathophysiological role in neurodegenerative disease. Neuropharmacology 45, 14–44. 10.1016/s0028-3908(03)00140-0 12814657

[B7] Fornes O. Castro-Mondragon J. A. Khan A. van der Lee R. Zhang X. Richmond P. A.. (2020). JASPAR 2020: update of the open-access database of transcription factor binding profiles. Nucleic. Acids Res. 48, D87–D92. 10.1093/nar/gkz1001 31701148 PMC7145627

[B8] Giasson B. I. Duda J. E. Quinn S. M. Zhang B. Trojanowski J. Q. Lee V. M. (2002). Neuronal α-synucleinopathy with severe movement disorder in mice expressing A53T human α-synuclein. Neuron 34, 521–533. 10.1016/s0896-6273(02)00682-7 12062037

[B9] Green A. R. Youdim M. B. (1975). Effects of monoamine oxidase inhibition by clorgyline, deprenil or tranylcypromine on 5-hydroxytryptamine concentrations in rat brain and hyperactivity following subsequent tryptophan administration. Br J. Pharmacol. 55, 415–422. 10.1111/j.1476-5381.1975.tb06946.x 1203627 PMC1666694

[B10] Gu F. Chauhan V. Chauhan A. (2017). Monoamine oxidase-A and B activities in the cerebellum and frontal cortex of children and young adults with autism. J. Neurosci. Res. 95, 1965–1972. 10.1002/jnr.24027 28151561

[B11] Huang L. Frampton G. Rao A. Zhang K. S. Chen W. Lai J. M.. (2012). Monoamine oxidase A expression is suppressed in human cholangiocarcinoma *via* coordinated epigenetic and IL-6-driven events. Lab Invest. 92, 1451–1460. 10.1038/labinvest.2012.110 22906985 PMC3959781

[B12] Ip C. W. Klaus L. C. Karikari A. A. Visanji N. P. Brotchie J. M. Lang A. E.. (2017). AAV1/2-induced overexpression of A53T-α-synuclein in the substantia nigra results in degeneration of the nigrostriatal system with Lewy-like pathology and motor impairment: a new mouse model for Parkinson’s disease. Acta Neuropathol. Commun. 5:11. 10.1186/s40478-017-0416-x 28143577 PMC5286802

[B13] Jankovic J. Chen S. Le W. D. (2005). The role of Nurr1 in the development of dopaminergic neurons and Parkinson’s disease. Prog. Neurobiol. 77, 128–138. 10.1016/j.pneurobio.2005.09.001 16243425

[B14] Jia C. Qi H. Cheng C. Wu X. Yang Z. Cai H.. (2020a). α-Synuclein negatively regulates Nurr1 expression through NF-κB-related mechanism. Front. Mol. Neurosci. 13:64. 10.3389/fnmol.2020.00064 32477062 PMC7235291

[B15] Jia C. C. Li G. Jiang R. Liu X. Yuan Q. Le W.. (2020b). B-cell receptor-associated protein 31 negatively regulates the expression of monoamine oxidase a *via* R1. Front. Mol. Biosci. 7:64. 10.3389/fmolb.2020.00064 32426368 PMC7212379

[B16] Jiang H. Jiang Q. Liu W. Feng J. (2006). Parkin suppresses the expression of monoamine oxidases. J. Biol. Chem. 281, 8591–8599. 10.1074/jbc.M510926200 16455660

[B17] Jiang H. Ren Y. Yuen E. Y. Zhong P. Ghaedi M. Hu Z.. (2012). Parkin controls dopamine utilization in human midbrain dopaminergic neurons derived from induced pluripotent stem cells. Nat. Commun. 3:668. 10.1038/ncomms1669 22314364 PMC3498452

[B18] Kabayama M. Sakoori K. Yamada K. Ornthanalai V. G. Ota M. Morimura N.. (2013). Rines E3 ubiquitin ligase regulates MAO-A levels and emotional responses. J. Neurosci. 33, 12940–12953. 10.1523/JNEUROSCI.5717-12.2013 23926250 PMC6619730

[B19] Kalia L. V. Lang A. E. (2015). Parkinson’s disease. Lancet 386, 896–912. 10.1016/S0140-6736(14)61393-3 25904081

[B20] Kang S. S. Ahn E. H. Zhang Z. Liu X. Manfredsson F. P. Sandoval I. M.. (2018). Alpha-Synuclein stimulation of monoamine oxidase-B and legumain protease mediates the pathology of Parkinson’s disease. EMBO J. 37:e98878. 10.15252/embj.201798878 29769405 PMC6003650

[B21] Kolla N. J. Bortolato M. (2020). The role of monoamine oxidase A in the neurobiology of aggressive, antisocial and violent behavior: a tale of mice and men. Prog. Neurobiol. 194:101875. 10.1016/j.pneurobio.2020.101875 32574581 PMC7609507

[B22] Kruger R. Kuhn W. Muller T. Woitalla D. Graeber M. Kosel S.. (1998). Ala30Pro mutation in the gene encoding α-synuclein in Parkinson’s disease. Nat. Genet. 18, 106–108. 10.1038/ng0298-106 9462735

[B23] Lees A. J. Hardy J. Revesz T. (2009). Parkinson’s disease. Lancet 373, 2055–2066. 10.1016/S0140-6736(09)60492-X 19524782

[B24] Lesage S. Anheim M. Letournel F. Bousset L. Honore A. Rozas N.. (2013). G51D α-synuclein mutation causes a novel parkinsonian-pyramidal syndrome. Ann. Neurol. 73, 459–471. 10.1002/ana.23894 23526723

[B25] Lin X. Parisiadou L. Sgobio C. Liu G. Yu J. Sun L.. (2012). Conditional expression of Parkinson’s disease-related mutant α-synuclein in the midbrain dopaminergic neurons causes progressive neurodegeneration and degradation of transcription factor nuclear receptor related 1. J. Neurosci. 32, 9248–9264. 10.1523/JNEUROSCI.1731-12.2012 22764233 PMC3417246

[B26] Monzani E. Nicolis S. Dell-Acqua S. Capucciati A. Bacchella C. Zucca F. A.. (2019). Dopamine, oxidative stress and protein-quinone modifications in Parkinson’s and other Neurodegenerative diseases. Angew. Chem. Int. Ed. Engl. 58, 6512–6527. 10.1002/anie.201811122 30536578

[B27] Ou X. M. Chen K. Shih J. C. (2006). Monoamine oxidase A and repressor R1 are involved in apoptotic signaling pathway. Proc. Natl. Acad. Sci. U S A 103, 10923–10928. 10.1073/pnas.0601515103 16829576 PMC1544150

[B28] Polymeropoulos M. H. Lavedan C. Leroy E. Ide S. E. Dehejia A. Dutra A.. (1997). Mutation in the α-synuclein gene identified in families with Parkinson’s disease. Science 276, 2045–2047. 10.1126/science.276.5321.2045 9197268

[B29] Proukakis C. Dudzik C. G. Brier T. MacKay D. S. Cooper J. M. Millhauser G. L.. (2013). A novel α-synuclein missense mutation in Parkinson disease. Neurology 80, 1062–1064. 10.1212/WNL.0b013e31828727ba 23427326 PMC3653201

[B30] Reiter R. J. (1991). Pineal melatonin: cell biology of its synthesis and of its physiological interactions. Endocr. Rev. 12, 151–180. 10.1210/edrv-12-2-151 1649044

[B31] Saucedo-Cardenas O. Quintana-Hau J. D. Le W. D. Smidt M. P. Cox J. J. De Mayo F.. (1998). Nurr1 is essential for the induction of the dopaminergic phenotype and the survival of ventral mesencephalic late dopaminergic precursor neurons. Proc. Natl. Acad. Sci. U S A 95, 4013–4018. 10.1073/pnas.95.7.4013 9520484 PMC19954

[B32] Shih J. C. (1991). Molecular basis of human MAOA and B. Neuropsychopharmacology 4, 1–7. 2003865

[B33] Shih J. C. Chen K. Ridd M. J. (1999). Monoamine oxidase: from genes to behavior. Annu. Rev. Neurosci. 22, 197–217. 10.1146/annurev.neuro.22.1.197 10202537 PMC2844879

[B34] Spillantini M. G. Schmidt M. L. Lee V. M. Trojanowski J. Q. Jakes R. Goedert M. (1997). α-synuclein in Lewy bodies. Nature 388, 839–840. 10.1038/42166 9278044

[B35] Tanaka Y. Engelender S. Igarashi S. Rao R. K. Wanner T. Tanzi R. E.. (2001). Inducible expression of mutant α-synuclein decreases proteasome activity and increases sensitivity to mitochondria-dependent apoptosis. Hum. Mol. Genet. 10, 919–926. 10.1093/hmg/10.9.919 11309365

[B36] Ugun-Klusek A. Theodosi T. S. Fitzgerald J. C. Burte F. Ufer C. Boocock D. J.. (2019). Monoamine oxidase-A promotes protective autophagy in human SH-SY5Y neuroblastoma cells through Bcl-2 phosphorylation. Redox Biol. 20, 167–181. 10.1016/j.redox.2018.10.003 30336354 PMC6197572

[B37] Westlund K. N. Denney R. M. Rose R. M. Abell C. W. (1988). Localization of distinct monoamine oxidase A and monoamine oxidase B cell populations in human brainstem. Neuroscience 25, 439–456. 10.1016/0306-4522(88)90250-3 3399053

[B38] Wong Y. C. Krainc D. (2017). α-synuclein toxicity in neurodegeneration: mechanism and therapeutic strategies. Nat. Med. 23, 1–13. 10.1038/nm.4269 28170377 PMC8480197

[B39] Wu J. B. Yin L. Shi C. Li Q. Duan P. Huang J. M.. (2017). MAOA-dependent activation of Shh-IL6-RANKL signaling network promotes prostate cancer metastasis by engaging tumor-stromal cell interactions. Cancer Cell 31, 368–382. 10.1016/j.ccell.2017.02.003 28292438

[B40] Zarranz J. J. Alegre J. Gomez-Esteban J. C. Lezcano E. Ros R. Ampuero I.. (2004). The new mutation, E46K, of α-synuclein causes Parkinson and Lewy body dementia. Ann. Neurol. 55, 164–173. 10.1002/ana.10795 14755719

[B41] Zhu Q. S. Chen K. Shih J. C. (1994). Bidirectional promoter of human monoamine oxidase A (MAO A) controlled by transcription factor Sp1. J. Neurosci. 14, 7393–7403. 10.1523/JNEUROSCI.14-12-07393.1994 7996184 PMC6576875

